# Does Orientation of Full-Thickness Groin Grafts Affect Hyperpigmentation in Burn Contracture and Syndactyly Hands?

**Published:** 2014-01

**Authors:** Mohammad Motamedolshariati, Ezzatollah Rezaei, Maryam-Sadat Shakeri, Arash Beiraghi-Toosi

**Affiliations:** 1 Vascular and Endovascular Surgery Research Center, Mashhad University of Medical Sciences, Mashhad, Iran;; 2 Endoscopic and Minimally Invasive Surgery Research Center, Mashhad University of Medical Sciences, Iran;; 3 Medical student, Faculty of Medicine, Islamic Azad University of Mashhad, Mashhad, Iran;; 4 Surgical Oncology Research Center, Mashhad University of Medical Sciences, Mashhad, Iran

**Keywords:** Skin graft, Full-thickness, Hyperpigmentation

## Abstract

**BACKGROUND:**

Some grafts harvested from the groin area do not become hyperpigmented and in an individual with multiple pieces of grafts, the hyperpigmentation of the pieces may vary. This study evaluates the orientation of the inset of groin grafts according to their donor sites (superior-inferior) and its role in graft hyperpigmentation.

**METHODS:**

Patients with hand burn contracture or syndactyly who required at least 2 pieces of grafts were enrolled. In each patient, one piece of the graft was inset in the same direction of the orientation of the donor site and the other in the opposite direction. Six months after the operation, the pigmentation was scored by a subjective scaling from 1 to 5.

**RESULTS:**

Thirty-four fingers of 15 patients were included. The mean grade of pigmentation in grafts inset in the direction of the donor site was 3.00±1.118 and in those inset in the opposite direction was 2.88±1.409. This was not statistically significant.

**CONCLUSION:**

Our findings revealed that although the grafts inset in the opposite direction of the donor site, they were less pigmented. So orientation of full thickness groin grafts did not affect hyperpigmentation of the grafts in burn contracture and syndactyly hands.

## INTRODUCTION

Release of hand burn contractures generally leads to deficiency of skin that necessitates skin grafting. Release of syndactyly hands, especially when the webbing is beyond the proximal interphalangeal joint, usually requires skin grafting.^1^ Generally, there are two options for skin grafting: Full thickness skin grafts (FTSG) and split thickness skin grafts (STSG). STSG are more vulnerable to contractures than FTSG^2^^-^^5^ and are more likely to require revisions.^6^^-^^8^ Although the results of studies are not consistent.^9^ The donor site of the STSG may be more annoying to the patient than the FTSG that is closed primarily. On the other hand, FTSGs may become hyperpigmented and hair growth may be an annoying problem with them.^10^^-^^13^ Theoretically, from the view of both long term function and appearance, the best option would be a FTSG without hyperpigmentation and hair growth.

The most popular donor site for the harvest of the FTSG is the groin area. The groin area is a good source for large grafts, it is easily closed primarily, and its scar is inconspicuous. Full thickness graft harvest from the groin is almost easy. On the other hand, FTSG harvested from the groin is vulnerable to hyperpigmentation and hair growth. In practice, some FTSGs harvested from the groin area do not become hyperpigmented. Even in an individual patient who needs multiple pieces of graft, some pieces of FTSGs do hyperpigment and others do not. FTSGs used in the hand are usually in small pieces. During their inset, the orientation of the grafted skin according to its original site (superior-inferior) is not mentioned. This may be a potential cause for varied hyperpigmentation. This study is planned to examine whether the orientation of the inset of FTSGs harvested from the invaluable donor site of the groin, according to their donor sites, has a role in the graft hyperpigmentation. 

## MATERIALS AND METHODS

Patients with burn contracture of fingers or syndactyly who required at least 2 pieces of FTSGs were included in the study. All the grafts were harvested elliptically from the groin area by the first, second and the last authors with a No.15 scalpel while the assistant exerted counter traction over the skin. After harvest, the graft was defatted and special attention was made to remove all hair follicles from the dermis of the graft. Each patient was the control of herself/himself. In every patient, one piece of the graft was inset in the same direction of the orientation of the donor site and the other piece in the opposite direction. The orientation of the graft in each finger was randomly determined before the operation and was not affected by the conditions of the operation. The grafts were inset with the similar tensions. Grafts were covered with tie-over dressings that were remained in their place for 5 days. Among the patients operated in the period between December 2009 to June 2011, 34 fingers (17 fingers grafted in the direction of the donor site, 17 fingers grafted in the opposite direction of the donor site) in 15 patients were studied. Grafts were checked according to the pigmentation by the third author 6 months after the operation while she was blind about the orientation of the grafts. The pigmentation of each piece of the graft was scored by a subjective scaling from 1 to 5 in which 1 was very mild pigmentation and 5 were very severe pigmentation cases. Comparison of the mean grade of hyperpigmentation was done with Mann-Whitney U test and the statistical significance was set at a *P* value of <0.05.

## RESULTS

**Fig. 1:**
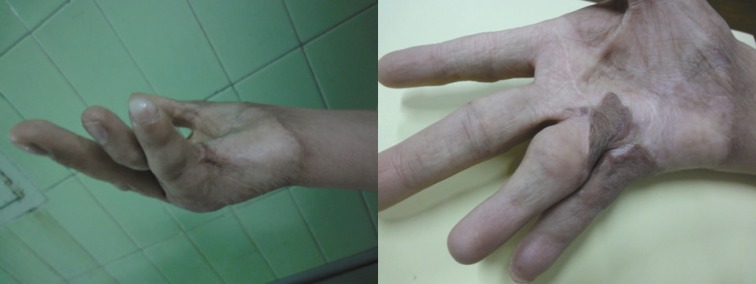
Burn contracture in a 24-year-old girl: the FTSG in the 4th finger was inset in the direction of the donor site and the 5th finger in the opposite direction. Six months following the operation, the graft in the 4th finger is more hyperpigmented

Fifteen patients were enrolled in the study. In these patients, 34 fingers were studied. The average age of the 15 patients was 17.4 years (min=1 yr, max=40 yrs). Eight patients were female and 7 were male. The pathologic process necessitating grafting in the fingers was burn in 11 patients and syndactyly in 4 patients (Figure 1). 

In one patient, the index and the middle fingers of one hand were compared with each other and the ring and little fingers of the same hand were compared separately. In another patient, 2 fingers of one hand were compared with each other and 2 fingers of the other hand were compared separately. In conclusion, we had 34 cases: 17 grafts that were inset in the direction of the donor site and were compared with 17 grafts that were inset in the opposite direction of the donor site. In the total 34 cases, the pathologic process was burn in 26 cases (76.5%) and syndactyly in 8 cases (23.5%). 

The mean grade of the pigmentation of the grafts that were inset in the direction of the donor site was 3.00±1.118 and the mean grade in the grafts that were inset in the opposite direction of the donor site was 2.88±1.409. The mean grade of hyperpigmentation was not significantly different in the two groups (*P*=0.734).

## DISCUSSION

Full-thickness skin grafting is a valuable treatment for release of finger burn contractures and syndactylies. On the other hand, hyperpigmentation of full-thickness grafts is an annoying problem to the patient. It does not occur uniformly in all patients and we cannot predict its occurrence and its severity before the operation. 

The only factor that is known to influence hyperpigmentation of the skin grafts is the site of the donor area. This has led to search for other donor sites such as upper arm, ulnar aspect of the wrist,^14^ plantar instep,^15^^-^^23^ and so on.^24^^-^^26^ All of these alternative sites have some disadvantages; the most common of them are poor scars and shortage of the available skin. Treatment of hyperpigmented palmar grafts is discussed in some studies.^16^^,^^26^^,^^27^ But, the prevention is better than the treatment.

Every effort should be done to find the factors related to the occurrence of hyperpigmentation and to decrease its chance or its severity. Orientation of some types of grafts is very important, e.g., vein and nerve grafts.^28^^-^^30^ But, to our knowledge, there is no study about the effect of the orientation of the skin grafts. 

In this study, it was shown that although the grafts inset in the opposite direction of the donor site were less pigmented, the difference was not statistically significant. But, it opens a search for identifying factors that may have a role in the hyperpigmentation of the grafts and probably predicting or decreasing its occurrence or severity. In conclusion, the orientation of full thickness groin grafts does not affect hyperpigmentation of the grafts in burn contracture and syndactyly hands.

## CONFLICT OF INTEREST

The authors declare no conflict of interest.

## Footenote

Please cite this paper as:

Motamedolshariati M, Rezaei E, Shakeri MS, Beiraghi-Toosi A. Does Orientation of Full-Thickness Groin Grafts Affect Hyperpigmentation in Burn Contracture and Syndactyly Hands? World J Plast Surg 2014;3(1):35-38.
